# Signaling Enzymes and Ion Channels Being Modulated by the Actin Cytoskeleton at the Plasma Membrane

**DOI:** 10.3390/ijms221910366

**Published:** 2021-09-26

**Authors:** Filip Vasilev, Yulia Ezhova, Jong Tai Chun

**Affiliations:** 1 Centre de Recherche du Centre Hospitalier de l’Université de Montréal (CRCHUM), 900 Rue St Denis, Montreal, QC H2X 0A9, Canada; 2 Maisonneuve-Rosemont Hospital Research Centre, University of Montreal, Montreal, QC H1T 2M4, Canada; yulia.ezhova@umontreal.ca; 3 Department of Biology and Evolution of Marine Organisms, Stazione Zoologica Anton Dohrn, 80121 Napoli, Italy

**Keywords:** actin cytoskeleton, phospholipase C, Src family kinase, Ca^2+^ signaling, PIP2, actin-binding protein, immune response, oocytes, epithelial cells, neurons

## Abstract

A cell should deal with the changing external environment or the neighboring cells. Inevitably, the cell surface receives and transduces a number of signals to produce apt responses. Typically, cell surface receptors are activated, and during this process, the subplasmalemmal actin cytoskeleton is often rearranged. An intriguing point is that some signaling enzymes and ion channels are physically associated with the actin cytoskeleton, raising the possibility that the subtle changes of the local actin cytoskeleton can, in turn, modulate the activities of these proteins. In this study, we reviewed the early and new experimental evidence supporting the notion of actin-regulated enzyme and ion channel activities in various cell types including the cells of immune response, neurons, oocytes, hepatocytes, and epithelial cells, with a special emphasis on the Ca^2+^ signaling pathway that depends on the synthesis of inositol 1,4,5-trisphosphate. Some of the features that are commonly found in diverse cells from a wide spectrum of the animal species suggest that fine-tuning of the activities of the enzymes and ion channels by the actin cytoskeleton may be an important strategy to inhibit or enhance the function of these signaling proteins.

## 1. Introduction

The dense network of the actin cytoskeleton intimately associated with the plasma membrane plays both structural and functional roles in animal cells. As the name ‘cytoskeleton’ implies, it confers rigidity to the cell membrane for mechanical protection. However, the actin cytoskeleton is not a static structure but undergoes constant remodeling in a living cell. The latter process is accelerated upon the arrival of cell signaling cues. The dynamic self-reorganizing nature of the actin cytoskeleton not only provides cell motility in certain cases, but also enables the cell to swiftly change its morphology to adapt to the fleeting physiological needs [1,2,3,4]. For example, platelets take the form of a biconvex discoid when circulating inside blood vessels, but when they are activated to make blood clots, drastic membrane projections take place due to actin polymerization and bundling on the cell surface [5]. On the other hand, in neuronal growth cones, polymerization dynamics of actin filaments underneath the plasma membrane contributes to the neurite’s pathfinding [6]. The enormous plasticity of cell surface topography due to the actin-mediated membrane protrusion and retraction is also manifested by phagocytic immune cells and fertilized eggs [7,8]. The rapid reorganization of the subplasmalemmal actin cytoskeleton in these cells assists in engulfing the foreign objects and fertilizing sperm, respectively. Thus, for a variety of cell types, remodeling the subplasmalemmal actin cytoskeleton is a fundamental part of the cell’s repertoire in dealing with diverse biological events taking place on the cell surface. 

In a living cell, the actin cytoskeleton is carved by a host of actin-binding proteins (ABPs) that bind, twist, sever, branch, or cap actin filaments (e.g., cofilin, gelsolin, villin, and so on). The activities of ABPs are modulated by various cell signals that trigger changes in their phosphorylation status, cytosolic pH, and intracellular Ca^2+^ concentration [9,10,11]. Moreover, ABPs are also regulated by phosphoinositide, a minor component of the plasma membrane that serves as a signaling molecule. For example, phosphatidylinositol 4,5-bisphosphate (PIP2), which is enriched in the inner leaflet of the plasma membrane lipid bilayer, interacts with most ABPs and thereby controls their activities and subcellular localization. Thus, a swift increase or decrease in PIP2 concentration in the specific places of the plasma membrane can serve as a second messenger signal that shifts the balance of the ABPs pools to activate or inhibit certain ABPs. This results in remodeling of the actin cytoskeleton [12,13,14]. 

A growing body of evidence suggests that the subplasmalemmal actin cytoskeleton affects chemical and mechanical signal transduction by casting a unique microenvironment in which the activities of certain signaling molecules are modulated by their relationship with the actin filaments undergoing dynamic changes [15,16]. While transmembrane proteins such as ion channels reside in the plasma membrane, some intracellular signaling enzymes can translocate and adhere to the plasma membrane by forming covalent bonds with lipid anchors such as myristic acid, palmate, and farnesoic acid. This is the strategy taken by some signaling molecules such as members of Src family kinases and Ras family GTPase [17,18,19]. It is also known that Src can bind to actin filaments due to its Src homology 2 (SH2) domain [20,21,22]. Thus, signaling enzymes such as Src can translocate themselves from cytosol to the plasma membrane or to the actin cytoskeleton. On the other hand, in the tight space around the plasma membrane–cytoskeleton interface, ion channels and pumps are often in contact with or in the vicinity of actin filaments, raising the possibility that their distribution and activity can be modulated by the actin cytoskeleton [23,24,25,26,27,28]. In line with the earlier findings that enzymes such as phospholipase, lipid kinases, and phosphatases are linked to the cytoskeleton [15], these observations suggest that actin cytoskeleton may serve as a signaling platform on which various external signals are transduced to the downstream effectors. Hence, subtle changes in the actin cytoskeleton are expected to affect the activities of these signaling molecules. 

As mentioned earlier for the immune cells, neurons, and fertilized eggs, certain cell signals induce rapid reorganization of the actin cytoskeleton. Obviously, in this case, the actin cytoskeleton is a target of the signal transduction. Conversely, subtle specific changes in the actin cytoskeleton may also facilitate or impede transduction of the signals because a number of signaling molecules are physically associated with the actin cytoskeleton. For example, the universal second messenger Ca^2+^ can reorganize the actin cytoskeleton in a number of different pathways including the ones that involve Ca^2+^-dependent ABPs [29,30,31,32]. On the other hand, the actin cytoskeleton can affect mobilization of Ca^2+^ in several different ways. To begin with, actin has a strong affinity to Ca^2+^ and thereby serves as a Ca^2+^ buffer or a barrier to the diffusion of Ca^2+^ ions [33,34,35]. The actin cytoskeleton may also affect the activities of the enzymes synthesizing the Ca^2+^-mobilizing second messengers such as inositol 1,4,5-trisphosphate (InsP_3_), or modulate the activities of some ion channels and pumps that transport Ca^2+^ across the membrane [24,26,36,37,38]. Indeed, studies in the eggs of echinoderm such as starfish and sea urchin have demonstrated that alteration of the egg cortical actin cytoskeleton profoundly affects the intensity and spatiotemporal pattern of the intracellular Ca^2+^ signals that are produced in the maturing oocytes and fertilized eggs [39,40,41,42,43,44]. This phenomenon of actin-dependent modulation of Ca^2+^ signaling is not restricted to oocytes and eggs. It has been intermittently reported that the activities of the signaling enzymes and ion channels involved in Ca^2+^ signaling and other ion flux are closely linked to the actin cytoskeleton in a variety of cell types. In this communication, we have reviewed some of the key findings on the topic in an attempt to understand its significance and the molecular mechanisms underlying the phenomenon.

## 2. Modulation of the Ca^2+^ Signal Transduction by the Actin Cytoskeleton in the Cells of Immune Response 

### 2.1. T-Lymphocytes

**Figure 1:**
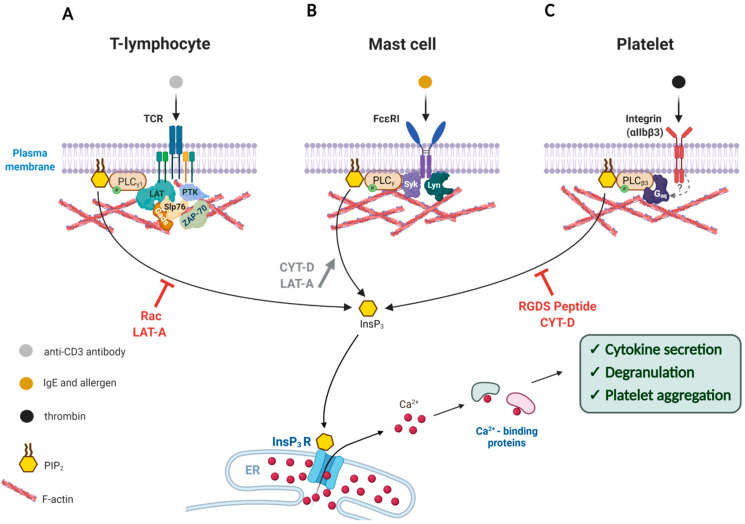
Modulation of the Ca^2+^ signal transduction by the actin cytoskeleton in the cells of immune response. Artificially altered actin dynamics during cell activation either enhances or represses Ca^2+^ signaling through the PLC/InsP_3_ pathway. (**A**) Exposure of T-lymphocytes to anti-CD3 antibody triggers T-Cell Receptor activation that leads to a cascade of downstream phosphorylation reactions that activates enzymes like PLC-γ1, ZAP-70 (Zeta-chain-associated protein kinase 70), PTKs as well as adaptor proteins such as LAT, Slp76, and Gads. Interference with the actin dynamics by use of Rac overexpression or Latrunculin-A (LAT-A) reduces phosphorylation of these enzymes, and thereby inhibits the PLC-γ1 activity and cytokine secretion. (**B**) Upon binding to antigen-IgE, the FcεRI receptor on the surface of a mast cell is phosphorylated and recruits more Lyn kinase to activate Syk kinase, which in turn stimulates PLC-γ to produce InsP_3_ and thereby release intracellular Ca^2+^ and trigger degranulation. If the concomitantly occurring actin polymerization is prevented by actin drugs LAT-A or Cytochalasin-D (CYT-D), the activation of PLCγ is even more enhanced. (**C**) Binding of thrombin to the integrin receptor (αIIbβ3) on the plasma membrane leads to enhanced polymerization of actin, which is accompanied by the translocation of PLCβ3 and other proteins. The resulting Ca^2+^ signal induces platelet aggregation. When the responsive actin polymerization is inhibited by CYT-D or fibrinogen antagonist tetra-peptide (RGDS), the extent of PLCγ activation and platelet aggregation is severely compromised. Note: The T-shaped red bars near the curved arrows denote inhibition.

Fine regulation of actin dynamics in the cell cortex is of great importance during the immune response of T cells. Signal transduction in T lymphocytes is often studied in Jurkat cells, which is an immortalized cell line derived from human T lymphocyte leukemia. These cells can be effectively activated to evoke Ca^2+^ signals and produce interleukin 2 (IL-2) by ligating its T cell co-receptor CD3 (cluster of differentiation 3) with the specific antibody [45]. Interestingly, when normal actin dynamics in Jurkat cells were disturbed by overexpressing the constitutively active or dominant negative form of small GTPase Rac, the cells often failed to respond correctly to the stimulation by the anti-CD3 antibody [46]. In this work, the cells transfected with Rac mutants produced a much reduced Ca^2+^ increase in response to the same activation. In addition, the stimulation by the anti-CD3 antibody did not significantly increase actin polymerization which is normally observed in the control cells. Remarkably, overexpression of the constitutively active Rac mutant (V12) led to a significant decrease in the enzyme activity of phospholipase C (PLC), as judged by PIP2 and InsP_3_ assays. According to the specific immunoprecipitation experiments followed by western blot analysis with anti-phosphotyrosine antibody, the extent of tyrosine-phosphorylation of PLC-γ1 and some other key proteins involved in signal transduction was significantly reduced by the expression of the Rac mutants. Since the PLC/InsP_3_ pathway is an important constituent of Ca^2+^ mobilizing machinery in T cells [45,47,48], the altered phosphorylation of PLC-γ1 is in part accountable for the alleviated intracellular Ca^2+^ response upon antigenic stimulation. In support of the idea that the compromised Ca^2+^ response in these Rac-transfected cells arise from the altered actin dynamics per se, and not from some unknown parallel effect of Rac mutants, it was demonstrated that a potent drug promoting actin depolymerization, Latrunculin-A (LAT-A), had a similar inhibitory effect on anti-CD3-induced Ca^2+^ response (Figure 1A). As intracellular Ca^2+^ signals in lymphocytes play an important role in the expression and secretion of cytokine, such changes might lead to compromised immune response [46,48,49,50]. 

### 2.2. Mast Cells 

The aforementioned findings in Jurkat T cells suggest that the cellular mechanism transducing the signals from the T cell receptor to intracellular Ca^2+^ increase may involve adaptive reorganization (polymerization) of the cortical actin cytoskeleton, and any interference with the latter process results in the reduction in the Ca^2+^ response. In other words, fine-tuning of the actin cytoskeleton beneath the plasma membrane is important for the generation of Ca^2+^ signals. This phenomenon is not restricted to T lymphocytes, although the exact mode in which the actin dynamics affects the intracellular Ca^2+^ signaling may vary from one cell type to another. For example, in Rat Basophilic Leukemia 2H3 (RBL-2H3) cells, which are often utilized as a model system for mast cells, filamentous actin (F-actin) has been shown to be implicated in intracellular signaling. Being a part of the immune system, mast cells are best known for allergic responses. The surface of mast cells is covered with immunoglobulin E (IgE) receptors (FcεRI), which have high affinity to the Fc region of IgE. Presenting allergens to the IgE-bound mast cells then leads to exocytosis of histamine and other contents (degranulation), which is dependent on both Ca^2+^ and the actin cytoskeleton [51,52]. Stimulating IgE-sensitized RBL-2H3 cells with an antigen causes an increase in F-actin assembly, InsP_3_ formation, and oscillatory Ca^2+^ increase in the cell. All these changes are considered to be essential for degranulation [53]. Interestingly, in the presence of Cytochalasin-D (CYT-D), another agent promoting actin depolymerization, the IgE-sensitized RBL-2H3 cells displayed a much reduced F-actin formation upon antigenic stimulation, but this cytoskeletal change paradoxically enhanced the response of the cells to the antigen by showing increased levels of InsP_3_ production and Ca^2+^ signals, which in turn led to increased degranulation [53]. Thus, actin depolymerization appears to have the opposite effects on T-cells (Jurkat) and mast cells (RBL-2H3) in terms of InsP_3_ production and Ca^2+^ signaling. The InsP_3_-boosting effect of CYT-D in stimulated RBL-2H3 cells was negated by the actin-stabilizing drug Jasplakinolide, suggesting that the enhancement of InsP_3_ and Ca^2+^ responses was the result of nothing but actin depolymerization. How does the actin cytoskeleton modulate the levels of InsP_3_ and Ca^2+^? Since U73122 (an inhibitor of PLC) also abolished the Ca^2+^-enhancing effects of CYT-D in the stimulated RBL-2H3 cells, it was proposed that PLC activation depends on the actin cytoskeleton, the reorganization of which accompanies FcεRI-induced tyrosine kinase activation. Now that the actin meshwork is loosened, PLC may be hyper-activated by antigenic stimulation (Figure 1B). Indeed, when actin polymerization was inhibited by LAT-A, the stimulated RBL-2H3 cells exhibited enhanced degranulation with higher PLC activity. In these cells, the extent of tyrosine-phosphorylation was also increased on the FcεRI receptor itself by the action of non-receptor type protein tyrosine kinases such as Lyn and Syk (Figure 1, see mast cells) [54]. As the augmented degranulation induced by LAT-A was nullified when the cells were stimulated by a potent inhibitor of protein tyrosine phosphatase pervanadate [54,55], the effect of the actin depolymerization appears to be directed to protein tyrosine kinases, PLC, and other molecules involved in the downstream pathway of FcεRI receptor. Interestingly, these actin drugs did not directly affect the store-operated Ca^2+^ entry or thapsigargin-induced Ca^2+^ release from the intracellular stores, suggesting that the effect of the actin cytoskeleton on the Ca^2+^ toolkits in this particular cell type is specifically on the PLC pathway. Hence, depolymerization of F-actin in the antigen-stimulated FcεRI in RBL-2H3 cells appears to promote PLC activation, whereas its excessive polymerization inhibits degranulation [53,56]. In support of the idea, another example can be taken. Degranulation in RBL-2H3 cells, to a lesser extent, can be initiated through antibody-mediated aggregation of the cell surface glycoprotein Thy-1 within the lipid rafts [57]. Interestingly, the presence of LAT-A again elicited more potent degranulation, following quicker and larger Ca^2+^ response. It is noteworthy that LAT-A treatment alone slightly increased the phosphorylation level of Syk and the enzymatic activity of phosphatidylinositol 3-kinase (PI3K), albeit without initiating degranulation [58]. Thus, the polymerization status of the actin meshwork near the plasma membrane appears to set the tone to degranulation in mast cells [58] and lymphocytes [46] by modulating the activities of the key signaling enzymes that promote Ca^2+^ increase and secretory response.

Stimulating IgE-sensitized mast cells (RBL-2H3) with antigens causes tyrosine-phosphorylation of the cytoplasmic tails of the FcεRI β and γ subunits by Lyn. This, in turn, leads to the phosphorylation of several downstream effectors including PLC-γ (Figure 1B), which results in PLC activation, InsP_3_ formation, and oscillatory Ca^2+^ increase in the cytoplasm [59,60,61]. In stimulated RBL-2H3 cells, FcεRI is colocalized with Lyn and F-actin. Curiously enough, inhibiting actin polymerization with CYT-D sustained FcεRI in the phosphorylated state much longer, which is in part accountable for the enhanced degranulation [62]. Furthermore, enhanced phosphorylation was also found in the downstream effector Syk, which is in line with the substantial augmentation of the intracellular Ca^2+^ level [54,55,63]. One possible explanation for all of these observations may be that some unknown phosphatases are also part of the FcεRI/F-actin complex, and that these enzymes lose their access to FcεRI once F-actin is depolymerized.

### 2.3. Platelets

The third example where the actin cytoskeleton is intimately involved in signal transduction across the plasma membrane is found in the thrombin-stimulated aggregation of platelets, the anucleated blood cells derived from megakaryocytes [64,65,66]. Upon binding to the agonist such as collagen at the site of disrupted endothelium, the platelet-specific integrin (α_IIb_β3) activates a variety of downstream enzymes to initiate Ca^2+^ and phosphorylation signaling pathways that will change cell shape and consolidate platelet aggregation to promote blood clotting [67,68,69]. Curiously, some of these enzymes translocate to the actin cytoskeleton near the plasma membrane to remain active and regulate the metabolism of phosphoinositide. For example, the activities of the actin cytoskeleton-bound PI3K, phosphatidylinositol 4-kinase (PI4K), diacylglycerol (DAG) kinase, and PLC were significantly increased upon platelet activation [21]. Likewise, translocation to the actin signaling platform significantly increases the activity of the phosphatidylinositol 4-phosphate 5 kinase C (Ptdins 4-P 5-kinase C) to enhance the synthesis of PIP2. The consequent shift of the balance in PIP2 level, in turn, is likely to induce remodeling of the actin cytoskeleton by way of the ABPs that have affinity to PIP2 [13,70]. Similarly, inositol polyphosphate 4-phosphatase is also translocated to the actin cytoskeleton following platelet activation [71,72]. Thus, the metabolism of phosphoinositide appears to be closely linked to the subplasmalemmal actin cytoskeleton to have a mutual influence on each other. On the other hand, activated integrins induce translocation of PLCβ3 to the actin cytoskeleton, which appears to require not only actin polymerization, but also phosphorylation of certain proteins, as judged by the fact that CYT-D or genistein (an inhibitor of tyrosine kinases) prevents the translocation [73]. In addition, blocking integrin by applying fibrinogen antagonist tetra-peptide (RGDS) or the inhibitory monoclonal anti-integrin β3 antibody prevents the thrombin-mediated enhancement of actin polymerization, which leads to failed translocation of PLCβ3 to the actin-rich region. As a result of deregulated actin polymerization, thrombin-induced platelet aggregation is considerably inhibited (Figure 1C) [73]. While PLCβ3 plays decisive roles in mobilizing intracellular Ca^2+^ (via InsP_3_) and in remodeling F-actin to induce platelet aggregation [74], its spatial dispatching in association with F-actin may signify another layer of control for the enzyme activity.

## 3. Modulation of Cytokine or Trophic Factor Signaling Pathways by the Cortical Actin Cytoskeleton

Constituting about 80% of the liver mass, hepatocytes are known to regenerate actively in a growth factor and cytokine-dependent manner [75]. When these cells are stimulated by epidermal growth factor (EGF), which binds to its receptor (EGFR) on the cell surface, an intracellular Ca^2+^ increase is induced in a pathway involving phosphorylation of PLC-γ1. However, primary cultures of hepatocytes with prolonged exposure to EGF (1–24 h) enter a refractory phase, during which the activation of PLC by EGF shows a rapid decline. Interestingly, this reduced effect of EGF on PLC was not attributable to decreased number of EGFR and PLC-γ1 per se, nor to reduced phosphorylation of EGFR and PLC-γ1 tyrosine phosphorylation, which did not change much during this period [76]. This apparent downregulation of PLC activity arose rather from the reduced localization of PLC-γ1 in association with the cortical actin cytoskeleton. Indeed, loosening up the actin meshwork with CYT-D not only restored InsP_3_ formation and Ca^2+^ mobilization back to the levels in the freshly isolated cells being exposed to EGF, but also increased colocalization of PLC-γ1 with the actin cytoskeleton. Thus, for the enzyme activity, its cytoskeletal context matters profoundly. In this case, it is quite remarkable that the rates of cytoskeletal reorganization and the consequent redistribution of PLC-γ1 can highly influence PLC-γ1 activity regardless of its phosphorylation status [76]. 

A similar phenomenon was observed when bone marrow-derived macrophage precursors were stimulated by macrophage colony stimulated factor (M-CSF) in a physiological model of macrophage differentiation [77]. Upon the cell’s exposure to M-CSF, PLC-γ2 in these cells readily translocates from the perinuclear zone to the cell periphery (plasma membrane) in a cytoskeleton-dependent manner. The translocated PLC-γ2 molecules tend to be tyrosine phosphorylated. As PLC-γ2 phosphorylation mainly depends on non-receptor type protein-tyrosine kinase Src, it is presumed that the receptor for M-CSF, which is a tyrosine kinase, phosphorylates Src and other downstream effectors. On the other hand, PLC-γ2 binds directly to actin through its SH2 domain, which is facilitated by the polymerization of actin in response to M-CSF [77]. However, it is not known whether the tyrosine-phosphorylation and translocation of PLC-γ2 in this process actually leads to the activation of the enzyme to induce InsP_3_ production and intracellular Ca^2+^ signals.

Furthermore, activity of Src itself appears to be modulated by the actin cytoskeleton. The growth cone of a neuron is filled with dynamically remodeling actin filaments that are searching for the right direction for axonal growth, following the guidance cues such as Netrin-1 (attractant) and Slit-2 (repulsive) [78,79,80,81]. Binding of these guidance cue molecules to the local cell surface receptors evokes a cascade of downstream pathway that involves tyrosine-protein kinases. Studies in neurons of sea slug *Aplysia californica* have shown that Src plays an important role in the growth cone dynamics by acting on cortactin and other substrates [82,83]. In the growth cone of developing rat brain, it has been shown that Src associated with the cytoskeleton has an intriguingly higher enzyme activity than the soluble ones [22]. When Src is phosphorylated on tyrosine-527, this negatively charged residue binds to the SH2 domain of Src itself, which induces conformational change of the enzyme so that it falls off from the cytoskeleton, thereby causing a decline in the enzymatic activity [22]. Therefore, association with the cytoskeleton in the cell cortex may serve as a platform modulating the activity of signaling enzymes. While Src in the growth cone of the Aplysia neuron colocalizes with both F-actin and microtubules, it is an open question whether Src binds to F-actin directly as its related protein Shc (Src homologous and collagen) does, or indirectly through other actin-binding proteins such as cortactin [84,85,86].

## 4. Actin-Binding-Proteins in Controlling Enzyme Activity

**Figure 2:**
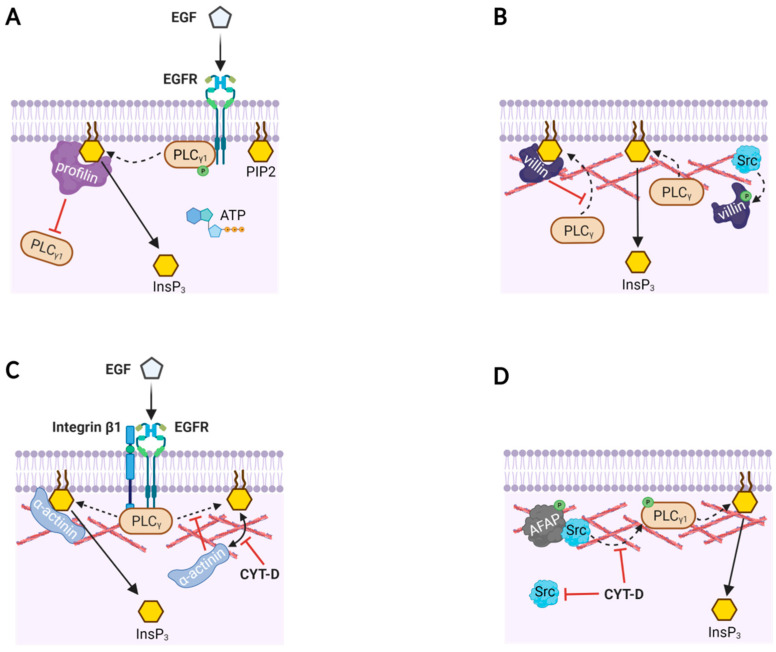
Actin-binding proteins modulate PLC activities with or without actin filaments. (**A**) A model based on in vitro reaction mixture containing phospholipid bilayer with PIP2, purified EGFR, PLC-γ1, ATP, and profilin. Profilin binds to PIP2, and thereby prevents PLC-γ1 from accessing its substrate PIP2. When PLC-γ1 is phosphorylated by the activation of EGFR, the inhibition by profilin is overcome and PIP2 is hydrolyzed to produce InsP_3_ and DAG (not shown). (**B**) Likewise, villin masks PIP2 from PLC-γ, but phosphorylated villin (by Src) loses its binding affinity to PIP2. Then, PLC has access to PIP2 to produce InsP_3_. (**C**) The focal adhesion complex in human mammary epithelial (HME) cells comprises PLC-γ, integrin β1, α-actinin, F-actin, and EGFR. Binding of EGF to EGFR induces EGF receptor dimerization, phosphorylation of PLC-γ, and increased α-actinin loading on PIP2. Here, α-actinin’s binding to PIP2 facilitates its hydrolysis by PLC-γ, and consequently the production of InsP_3_ doubles. Disruption of the actin cytoskeleton with Cytochalasin-D (CYT-D) dissociates α-actinin from PIP2, and the EGFR-associated PLC-γ is unable to cleave the PIP2 not bound to α-actinin. (**D**) Actin filament-associated protein (AFAP) in rat fetal lung cells presents Src to PLC-γ1 via actin filaments so as to activate the enzyme. As a result, InsP_3_ production is increased, but the effect is inhibited by CYT-D.

If the actin cytoskeleton modulates signal transduction in the subplasmalemmal region, ABPs may well play crucial roles behind the scenes. As its name implies, ABP binds to actin monomer (G-actin) or F-actin and influences the polymerization dynamics to remodel the actin cytoskeleton, often in an accelerated manner. Importantly, most ABPs have a stretch of basic amino acid residues that are thought to enable ABPs to anchor to PIP2 on the inner leaflet of the plasma membrane [13]. Their competitive binding to both actin and PIP2, or even directly to enzymes, would have a profound impact not only on the actin cytoskeleton dynamics, but also on signal transmission [87,88]. According to an in vitro study with purified proteins and synthetic membranes, when profilin (an actin monomer-sequestering protein) is bound to PIP2, it prevents PIP2 from being cleaved by non-phosphorylated quiescent PLC-γ1. However, upon activation of EGFR and the subsequent tyrosine phosphorylation of PLC-γ1, the inhibitory binding of profilin to PIP2 is overcome by the phosphorylated PLC-γ1, and PIP2 is cleaved (Figure 2A). Profilin binding to PIP2 not only inhibits its own interaction with actin [70,89], but may also prevent non-phosphorylated PLC-γ1 from binding its substrate PIP2 and thereby impede the formation of the second messengers InsP_3_ and DAG. Thus, binding or displacement of profilin on PIP2 may be a critical factor controlling the activity of certain enzymes that interact with PIP2 [89]. In this case, what influences the enzyme activity of PLC-γ1 is not the actin filaments per se, but the accessary protein that binds to actin. 

ABP can also act as a converging point for the transduced signals. A multifunctional ABP villin, which severs, caps, nucleates, and bundles F-actin, can directly bind to PLC-γ1 and thereby affect the enzyme activity in the subplasmalemmal cell compartments [90]. In vitro studies using recombinant and native villin purified from the brush borders of chicken intestinal epithelial cells demonstrated its interaction with PLC-γ1 and its substrate PIP2. Villin’s binding to PIP2 inhibits PLC-γ1, but villin’s tyrosine-phosphorylation by Src reverses this inhibition because phosphorylated villin no longer binds to PIP2 (Figure 2B). As villin is tyrosine phosphorylated in vivo in response to receptor activation and also binds to the membrane-bound PLC-γ1 and Ca^2+^ [91,92], the multifunctional ABP villin is likely to regulate the actin cytoskeleton dynamics and converge signal transduction pathways. Since PIP2 is predominantly located in the plasma membrane [93,94], all these interactions among PIP2, actin, and ABPs are the molecular events taking place in the tight junction between plasma membrane (i.e., PIP2) and the subplasmalemmal actin cytoskeleton. 

Besides interacting with PIP2, ABPs are often found as a component of a receptor complex. In human mammary epithelial (HME) cells, immunoprecipitation of EGFR-bound proteins detected α-actinin (a Ca^2+^-dependent F-actin bundling protein) together with PLC-γ, actin, and integrin β1 in the adhesion complex even before the EGF stimulation [95]. When the cells are treated with CYT-D, they retract and change their shapes. Even so, the interaction in the adhesion complex between the EGFR and PLC-γ was not affected, implying a direct interaction between the two. Upon EGF stimulation, however, CYT-D inhibited PIP2 hydrolysis, and decreased the level of EGF-induced α-actinin binding to PIP2. Thus, the polymerization status of the actin cytoskeleton appears to affect the EGFR complex only when it is in the stimulated state. The diminished PLC activity following artificial depolymerization of F-actin by CYT-D might be attributable to the changed subcellular location of α-actinin departing from the PIP2-bound pool. Interestingly, in concert with other ABPs such as myosin, α-actinin seems to have an opposite effect on the PLC activity. While PIP2-bound profilin and villin inhibit PIP2 hydrolysis, α-actinin bound to PIP2 in the EGF-stimulated HME cells facilitates hydrolysis of PIP2 by PLC-γ (Figure 2C) [95,96]. 

An example of how ABP binding to F-actin mediates cell signaling is found during the stretch-induced proliferation of rat fetal lung cells [97]. Mechanical stretch induces cytoskeletal deformation, which evokes signal transduction by translocating Src from cytosol to the actin cytoskeleton. This process is mediated by the binding of Src to actin filament-associated protein (AFAP) adaptors that localize on and cross-link the actin fibers. This binding promotes Src activation and tyrosine phosphorylation of AFAP and other target proteins of Src (e.g., phospholipase C-γ, Figure 2D) [97]. Hence, the actin cytoskeleton in the cell cortex takes part in transducing not only extracellular chemical cues, but also the external physical forces into intracellular biochemical signals by activating a non-receptor tyrosine-protein kinase Src. 

## 5. Modulation of the Ion Channels Activity by the Actin Cytoskeleton 

As the actin cytoskeleton is in vicinity to the plasma membrane and organelles, the ion channels residing on the cell membranes are surrounded by a meshwork of actin filaments. More often than not, some of the ion channels are physically in contact with actin. Given that the actin cytoskeleton is constantly reorganizing itself and rapidly changes its spatial arrangement following the cell signaling cues, it is conceivable that certain configurations of actin cytoskeleton near the ion channels may either favor or inhibit opening of the channel [98]. For example, studies on mouse mammary adenocarcinoma cells expressing human cystic fibrosis transmembrane conductance regulator (CFTR, a phosphorylation and ATP-gated anion channel) have shown that brief exposure to CYT-D significantly enhances the channel conductance for Cl^-^ in the current–voltage curve to the extent that is comparable to the time when the channel is activated by adenosine 3’,5’-cyclic monophosphate (cAMP), the second messenger activating protein kinase A (PKA) [99]. On the other hand, a more extensive disassembling of the actin filaments by longer exposure to CYT-D (6 to 9 h) completely abolished the channel activity including the cAMP-induced currents. In the same condition, addition of PKA to the cytoplasmic side of the excised cell patches did not restore the channel activity either. Remarkably, however, addition of exogenous actin and ATP (to stimulate de novo polymerization) restored it [99]. Thus, while moderate shortening of long actin filaments activates the channel, excessive depolymerization actually antagonizes the channel activity in this particular case. Furthermore, CFTR activity was inhibited either by disturbing polymerization/depolymerization balance by preventing polymerization with deoxyribonuclease I (DNase I, also an ABP) [100,101] or by inducing actin bundling with filamin [99]. 

Modulation of ion flux by the actin cytoskeleton is not restricted to anion channels. Indeed, the polymerization status of cortical actin also affects Na^+^ channel activity in epithelial cells. In the Xenopus kidney A6 cell line, Na^+^ channels are co-localized with F-actin [102]. Moreover, addition of CYT-D to the excised patch showing no spontaneous channel activity suddenly induced the Na^+^ current within 1–2 minutes. Similarly, addition of G-actin at a concentration that stimulates the formation of short actin filaments activated the Na^+^ channel within five minutes. In contrast, adding long actin filaments preassembled in vitro did not have such an effect, suggesting that the length of the actin filaments is critical for the Na^+^ channel activation. In the given experimental system, it thus appears that F-actin of an intermediate length is favorable in promoting the activity of CFTR and Na^+^ channel activities. 

The actin cytoskeleton also affects the activity of voltage-gated ion channels in excitable cells. Disruption of the actin cytoskeleton in salamander retinal ganglion cells can alter the activity of both L-type Ca^2+^channels and K^+^ channels [103]. Measurements of the whole-cell currents demonstrated that the addition of actin-depolymerizing drugs (LAT and CYT) substantially reduced the peak of the Ca^2+^ current, whereas the same treatment inhibited the sustained outward K^+^ currents elicited by depolarizing pulses. The effect was attributed to the changes in the actin cytoskeleton per se because it was all nullified by adding phalloidin (a drug stabilizing F-actin) in the patch pipette. Consistently, LAT-A inhibited the Ca^2+^ current produced by elevated KCl concentration, a less-invasive method to depolarize the membrane, and the potency of inhibition was proportional to the length of LAT-A exposure [103]. These observations imply that excessive actin depolymerization interferes with the normal ion channel function and thereby inhibits ion fluxes across the plasma membrane. Furthermore, neurons of rat dorsal root ganglion (DRG) exposed to LAT-A during the patch-clamp recordings exhibited multiple action potential firings with increased duration, drawing a drastic contrast with the control cells that showed only one action potential in response to the same depolarizing current [104]. Under the voltage-clamp condition, LAT-A inhibited K^+^ current in a dose-dependent manner. This finding implies that LAT-A-induced actin depolymerization may impede repolarization of the membrane potential so that triggering multiple action potentials is facilitated. Again, phalloidin alleviated both the LAT-A-induced inhibition of K^+^ current and the frequency-modulating effect on the action potentials, corroborating that the effects were because of the alteration of F-actin [104]. In this experiment, actin drug inducing depolymerization altered neither the resting potential nor the amplitude of the action potentials, but specifically inhibited the voltage-activated Ca^2+^ current. Hence, actin depolymerization dramatically increases the electrophysiological excitability of DRG neurons, whereas the similar condition in salamander retinal ganglion cells appears to have an inhibitory effect on the activities of voltage-gated Ca^2+^ channels.

It is noteworthy that the actin cytoskeleton also interacts with Ca^2+^ pumps and thereby modulates their activities. For example, Ca^2+^ pumps at the plasma membrane (plasma membrane Ca^2+^ ATPase, PMCA) extrude Ca^2+^ ions to the extracellular space against the concentration gradient by spending ATP energy. Interestingly, the activity of PMCA is modulated by the actin filaments that interact with the pumps [24,26,105]. A similar modulation by the actin cytoskeleton has also been observed with other Ca^2+^ pumps present in the endoplasmic reticulum or secretary vesicles [106,107]. Since actin has strong affinity to both Ca^2+^ and ATP, a shift in F-actin’s polymerization dynamics is expected to interfere with the activity of the Ca^2+^ pumps. Nonetheless, the precise mechanism by which the actin cytoskeleton modulates the activity of Ca^2+^ channels and pumps is largely unknown.

## 6. F-Actin Depolymerization Is Often Sufficient to Trigger Cell Signaling 

It is noteworthy that, in the aforementioned study on DRG neurons, treatment of the cells with LAT-A alone produced intracellular Ca^2+^ increase in 65% of the cases [104]. It now merits reviewing some examples where cells exhibit spontaneous Ca^2+^ signals or activate certain signaling enzymes merely by the changes in the cortical actin cytoskeleton without the aid of physiological cues or trophic factors. The dense meshwork of F-actin in the crowded subplasmalemmal space could serve as an anchoring point or a platform on which certain signaling proteins can carry out their functions. On the other hand, the same cytoskeletal structure may instead act as a physical barrier for the signaling molecules [108,109]. When F-actin in the zone depolymerizes, PIP2 in plasma membrane is expected to become more accessible to ABPs and enzymes like PLC. According to this model, in certain cases, depolymerization of the actin cytoskeleton itself can trigger PLC-mediated PIP2 hydrolysis to generate InsP_3_ and intracellular Ca^2+^ signals even in the absence of physiological stimuli. One such example is B lymphocytes (B cells) where exposure to cytochalasins (A, B, D, and E) induces rapid and sustained increment of intracellular Ca^2+^ [110]. Although the Ca^2+^ derives largely from the extracellular medium, a small transient Ca^2+^ elevation is consistently observed when cells were kept in a Ca^2+^-free medium, indicating that some contribution is also made by Ca^2+^ mobilization from the internal stores [110]. In line with the latter finding, CYT-D elicits a more than twofold increase in intracellular InsP_3_ level in B cells within 5 minutes of incubation [111]. The same effect appears to be due to CYT-D’s specific action on actin because a similar molecule, chaetoglobosins C, which does not bind to actin, failed to do so. Hence, depolymerization of F-actin itself is likely to be the cause of InsP_3_ increase in B cells. Given that the artificial actin depolymerization triggering cellular responses (e.g. Ca^2+^ increase) is reminiscent of the antigen-induced B cell activation, rapid changes in actin dynamics by the external cues may launch multiple signaling pathways during B cell receptor activation and thereby play more important roles than was previously appreciated [112]. While the precise molecular mechanism underlying the Ca^2+^ increase evoked by actin depolymerization is not known, it has been shown that B cells obtained from the transgenic mice lacking stimulatory co-receptor CD19 (cluster of differentiation 19) do not respond to LAT-A with intracellular Ca^2+^ increase. This observation suggests that the Ca^2+^-mobilizing effect exerted by actin depolymerization is mediated by CD19. In support of the idea, the responsiveness to LAT-A was restored in the bone marrow chimera cells obtained by injecting exogenous bone marrow cells expressing some mutant forms of CD19. However, the cells expressing specific CD19 mutants unable to bind PLC or Fyn failed to respond to LAT-A with the expected Ca^2+^ increases [112]. These findings are compatible with the idea that F-actin not only regulates the location of B cell receptors and CD19 [113], but may also play a role in restraining spontaneous signaling in the resting B cells.

**Figure 3:**
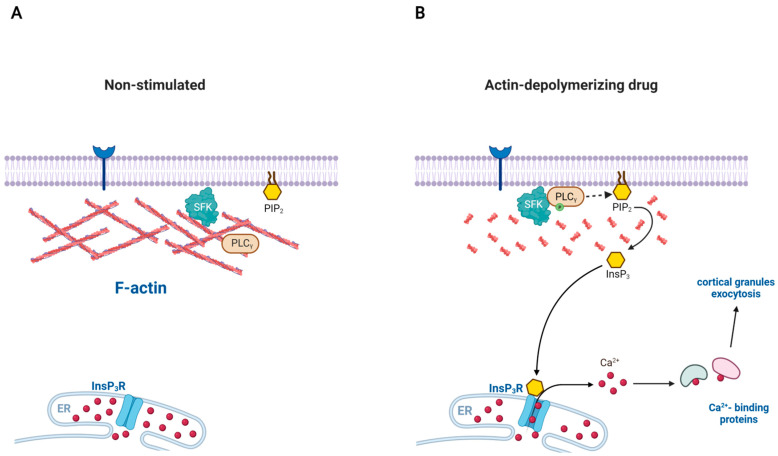
Disassembly of actin filaments can spontaneously activate PLC in certain cells: a model involving SFK. (**A**) Src family kinases (SFK) can be recruited to the plasma membrane due to their myristoylation, but the enzymes hardly have access to its substrate PLC-γ because of the actin cytoskeleton to which SFK is often associated. (**B**) When the actin cytoskeleton is disassembled, the physical barrier is essentially removed. SFK now gains access to PLC-γ and activates it. The phosphorylated PLC-γ now hydrolyzes the plasma membrane PIP2 to produce InsP_3_, a Ca^2+^-mobilizing second messenger acting on the receptor ion channel on the endoplasmic reticulum (ER). In the case of starfish egg (*Astropecten aranciacus*), disassembly of subplasmalemmal F-actin with Latrunculin-A and other drugs leads to increased production of InsP_3_ and the consequent rise of intracellular Ca^2+^ levels in the form of repetitive waves.

A similar phenomenon has also been reported in starfish eggs in which depolymerization of subplasmalemmal F-actin itself is sufficient to activate a signaling pathway to trigger intracellular Ca^2+^ increase and egg activation [38,114]. During meiotic maturation, the cortex and cell surface of starfish eggs are drastically reorganized in terms of vesicular structure and the subplasmalemmal actin network. These eggs readily respond to a fertilizing sperm with a sharp rise of InsP_3_ and intracellular Ca^2+^ increase. However, the Ca^2+^-mobilizing machinery of the egg is under tight control until the due signal (fertilization) arrives. This prohibitive mechanism preventing erratic triggering of Ca^2+^ signaling in unfertilized eggs is dependent upon the subplasmalemmal actin cytoskeleton. Indeed, when exposed to the drugs promoting actin depolymerization, eggs of certain starfish species (*Astropecten aranciacus*) ‘spontaneously’ exhibit intracellular Ca^2+^ increases in the absence of sperm (Figure 3). The initial finding with mature eggs of *Astropecten aranciacus* featured several interesting points: (i) LAT-A generates a Ca^2+^ wave that resembles the one elicited by the fertilizing sperm; (ii) the Ca^2+^ wave is often accompanied by Ca^2+^ influx forming cortical flash; (iii) the Ca^2+^ wave and influx recur for hours; (iv) the Ca^2+^ wave is blocked by heparin, the conventional inhibitor of InsP_3_ receptor; and (v) the ‘spontaneous’ sperm-free Ca^2+^ increase induced by LAT-A requires maturation of oocytes, which renders the cells more sensitive to InsP_3_ by an order of magnitude [114]. Thus, it appears that depolymerization of F-actin in these eggs generated Ca^2+^ waves in a pathway involving InsP_3_ receptor. However, this conclusion was obscured by the later finding that heparin drastically hyperpolymerizes actin in the subplasmalemmal region [40,115], and by the intriguing possibility that polymerization and depolymerization of actin could respectively serve as mechanisms by which to sequester and release intracellular Ca^2+^ inside cells due to actin’s strong affinity to Ca^2+^ [116]. If the latter scenario holds, LAT-A might have increased Ca^2+^ in a direct pathway independent of the InsP_3_ receptor. This very idea was put to the test in a recent study [38]. First of all, the LAT-A-induced Ca^2+^ wave and flux in *A. aranciacus* eggs was not due to some unknown side effects of the actin drug, but was a bona fide consequence of actin depolymerization. The Ca^2+^ waves triggered by LAT-A were replicated by other drugs similarly promoting actin depolymerization (i.e., cytochalasin B and mycalolide B), but inhibited by jasplakinolide and phalloidin, which stabilize actin filaments. The cytoskeletal changes induced by LAT-A did not make the eggs more sensitive to InsP_3_, but instead increased the rate of InsP_3_ synthesis as judged by the ELISA assay and the decrease of PIP2 on the plasma membrane. In agreement with this finding, the LAT-A-induced Ca^2+^ waves were severely suppressed by inhibitors of PLC (U73122, neomycin) and by the dominant negative recombinant protein containing the tandem SH2 domains of PLC-γ [38]. Interestingly, exposure of sea urchin (*Paracentrotus lividus*) eggs to LAT-A also led to a significant increase in InsP_3_. Taken together with the findings in the immune cells of mammals, these results suggest that subplasmalemmal region of animal cells manifesting enormous reactivity toward sperm or immunogenic elements are equipped with potent signaling toolkits such as components of the PLC/InsP_3_ signaling pathway that are under the tight controls of the actin cytoskeleton on the cell surface.

## 7. Concluding Remarks

In this study, we reviewed some of the experimental evidence that artificial modification of the actin cytoskeleton results in significant changes in the activities of certain enzymes and ion channels. The Ca^2+^ signaling pathway made of a cascade of steps involving cell surface receptors, Src family protein kinases, PLC, InsP_3_, and InsP_3_ receptor is one of the most extensively studied signal transduction mechanisms [117,118,119,120]. Not surprisingly, the findings in this topic have largely centered on the actin-dependent modulation of the enzymes or channel activities related to intracellular Ca^2+^ signaling via the PLC/InsP_3_ pathway. Nonetheless, it is noteworthy that such a phenomenon has been observed in a variety of cell types from diverse phyla (Table 1), which suggests that the physical and functional association of the enzymes and ion channels with the actin cytoskeleton is a physiologically and evolutionally significant strategy of cells. On the other hand, relatively less is known about similar physical and functional links to other forms of cytoskeleton, although it has been intermittently reported that some metabolic and signaling enzymes are associated with microtubules and intermediate filaments [121,122,123]. 

The ability to bind to cytoskeletal filaments provides the enzymes and ion channels with an advantage in trafficking and precise subcellular positioning. The enzymes’ binding to actin results in mutual influence, as exemplified by DNase I; actin suppresses DNase I activity, while DNase I in turn inhibits actin polymerization [100,101]. Furthermore, polymerization and depolymerization kinetics add nuance to the relationship between the enzymes and its substrates or binding partners comprising the enzyme complex. With the change in the cytoskeleton, the distance between them may increase or decrease. Thus, the actin cytoskeleton may facilitate interactions between the signaling molecules that are attached to it. Conversely, it may also assist in preventing unintended contacts between signaling molecules by sequestering them. Spontaneous generation of Ca^2+^ signals by the disruption of the actin filaments supports the latter idea. Hence, the concept that the actin cytoskeleton modulates the activities of enzymes and ion channels might have physiological significance from several perspectives. If signaling molecules like PLC are linked to the actin cytoskeleton and their activities are modulated by polymerization and depolymerization status of actin in a variety of cell types and in a wide spectrum of animal species, would the phenomenon have physiological relevance that has not been appreciated much to date? Furthermore, it bears an emphasis that the cytoskeleton also contributes to a cell’s sensing and conduction of the mechanical signals. In theory, it is conceivable that contact or collision between cells in a situation like fertilization may transmit a mechanical signal that induces local rearrangement of actin filaments, which in turn generates a chemical signal to activate certain enzymes and channels. Such a cell signaling mechanism has not been much explored, but may merit formal investigations in the future. 

## Figures and Tables

**Table 1:** Effects of interference with actin dynamics on cell signaling.

Cell Type	Actin Modifier	Effect	Reference
Jurkat cell (T-lymphocyte)	Active Rac mutant	Reduces PLC-γ1 activity and Ca^2+^ responses to anti-CD3.	[46]
	Latrunculin-A	Reduces Ca^2+^ responses to anti-CD3.	
RBL-2H3 (mast cell)	Cytochalasin-D	Enhances InsP_3_ production and Ca^2+^ response via FcεRI pathway.	[53]
	Latrunculin-A	Increases PLC activity, FcεRI phosphorylation, and degranulation.	[54,62]
Platelets	Cytochalasin-D	Reduces PLCβ3 translocation to the actin cytoskeleton; inhibits platelets aggregation.	[73]
Hepatocytes	Cytochalasin-D	Increases colocalization of PLC-γ1 with the actin cytoskeleton; restores InsP_3_ production and Ca^2+^ response to EGF.	[76]
Human mammary epithelial cell	Cytochalasin-D	Decreases α-actinin’s binding to PIP2; inhibits PIP2 hydrolysis after EGF receptor activation.	[95]
Rat fetal lung cells	Mechanical force	Stimulates Src activity and its translocation to the actin cytoskeleton.	[97]
Mouse mammary adenocarcinoma cells (transfected)	Cytochalasin-D	Enhances human CFTR activity conducting Cl^-^ (10 min), abolishes CFTR activity (>6 h);	[99]
	DNase I	Inhibits actin-induced CFTR activity.	[99]
	Filamin	Inhibits actin-induced CFTR activity.	
Xenopus laevis A6 cells	Cytochalasin-D	Stimulates Na^+^ channel activity.	[102]
Salamander retinal ganglion cells	Cytochalasin-D, Latrunculin-A	Reduces L-type Ca^2+^ channel activity; Inhibits voltage-gated K^+^ channel activities.	[103]
Rat dorsal root ganglion	Latrunculin-A	Inhibits K^+^ current; increases frequency of action potential.	[104]
B lymphocytes	Cytochalasin-A,B,D,E	Induces Ca^2+^ influx and release from internal stores.	[110]
	Cytochalasin-D	Increases the intracellular InsP_3_ level.	[111]
Starfish egg (*Astropecten aranciacus*)	Latrunculin-A	Increases PLC-γ activity, InsP_3_ production, and intracellular Ca^2+^.	[38]
	Latrunculin-A, Cytochalasin-B, Mycalolide-B	Triggers intracellular Ca^2+^ releases and influx.	[38]
Sea urchin egg (*Paracentrotus lividus*)	Latrunculin-A	Increases InsP_3_ production.	[38]

## Abbreviations

CD3	Cluster of differentiation 3
PLC	Phospholipase C
PIP2	Phosphatidylinositol 4,5-bisphosphate
RBL-2H3	Rat Basophilic Leukemia 2H3
αIIbβ3	Platelet-specific integrin receptor
DAG	Diacylglycerol
EGF	Epidermal growth factor
M-CSF	Macrophage colony stimulated factor
HME	Human mammary epithelial
AFAP	Actin-filament associated protein
CFTR	Human cystic fibrosis transmembrane conductance regulator
PKA	Protein kinase A
CYT-D	Cytochalasin D
LAT-A	Latrunculin A
cAMP	adenosine 3’,5’-cyclic monophosphate
